# Genome-Wide Identification and Expression Analysis of Starch Biosynthesis-Related Gene Families in Wheat

**DOI:** 10.3390/ijms27093876

**Published:** 2026-04-27

**Authors:** Qinlong Zhao, Longjiao Hu, Xinye Wu, Bo Ma, Weining Song, Xiaojun Nie, Shuzuo Lv

**Affiliations:** 1 Luoyang Academy of Agriculture and Forestry Science, Luoyang Key Laboratory of Crop Molecular Biology and Germplasm Enhancement, Luoyang 471000, China; zhaoqlong@nwafu.edu.cn; 2 Hainan Research Institute, Northwest A&F University, Sanya 572025, China; small@nwsuaf.edu.cn; 3 State Key Laboratory of Crop Stress Resistance and High-Efficiency Production, College of Agronomy, Northwest A&F University, Yangling 712100, China; longjiaohu@nwafu.edu.cn (L.H.); xinye@nwafu.edu.cn (X.W.); 2079723465@nwafu.edu.cn (B.M.); sweining2002@yahoo.com (W.S.)

**Keywords:** wheat, starch synthesis-related genes, starch characteristics, expression profiles

## Abstract

Starch synthesis is critical for crop yield and quality and is regulated and coordinated by a series of key enzymes encoded by starch synthesis-related genes (SSRGs). Although this process is well characterized in many crops, the genomic location and expression patterns of SSRGs in wheat remain unclear. Here, we performed a genome-wide analysis and identified 78 SSRGs in wheat, classified into the AGPase, SSS, GBSS, SBE, and DBE subfamilies. SSRGs within each subfamily showed conserved motifs and domain organization. RNA-seq analysis indicated that most SSRGs are expressed during early grain development. We further examined genetic variation in SSRGs across wheat and its progenitors using re-sequencing data. Diploid wheat showed greater genetic differentiation and diversity than tetraploid and hexaploid wheat. Five SSRGs exhibited significant haplotype differences between emmer wheat and common wheat; emmer wheat displayed diverse haplotypes, whereas common wheat showed a single dominant haplotype. Finally, starch characteristics differed between emmer wheat and common wheat in amylose content and thermodynamic properties, while viscosity, crystal structure, and morphology were largely similar. Overall, this study systematically characterizes SSRGs in wheat and provides insights for improving starch quality.

## 1. Introduction

Starch, which constitutes 65–80% of wheat grain components, is the primary source of dietary calories for humans [1]. Beyond its nutritional role, it is also an important raw material in the chemical industry [2]. In the cereal endosperm, starch comprises amylose and amylopectin, and differences in their chain structures confer distinct physicochemical properties [3]. Although the starch biosynthetic pathway is largely conserved across plant species [4], variations in gene expression (within or between species), during starch granule formation lead to substantial differences in granule characteristics [5]. Starch biosynthesis in cereals involves five major enzyme classes: adenosine diphosphate glucose pyrophosphorylase (AGPase), granule-bound starch synthase (GBSS), soluble starch synthases (SSI, SSII, SSIII, and SSIV), starch debranching enzyme (DBE), and starch branching enzyme (SBE) [6,7,8,9]. AGPase catalyzes the formation of ADP-glucose, while GBSS elongates glucan chains using ADP-glucose, contributing to amylose synthesis [10]. In contrast, amylopectin is synthesized primarily through the coordinated actions of SBE and soluble starch synthases (SSS), although their relative contributions vary across species and tissues, resulting in structural diversity [11]. DBE, including pullulanase and isoamylase, further modifies glucan chains and plays a key role in amylopectin structural formation [12].

Monosaccharides in the grain are first assembled into nascent starch granules through coordinated enzymatic activity. These nascent starch granules subsequently serve as templates for the formation and elongation of amylose and amylopectin chains of varying lengths. Subsequent modification and organization of these chains by multiple enzymes generate the final starch architecture [13]. The resulting starch phenotype is determined by factors including total starch content, amylose-to-amylopectin ratio, chain length distribution and granule crystalline and morphological features. Together, these factors govern key functional properties, including viscosity, thermal behavior and extensibility [14,15].

Bread wheat (*Triticum aestivum* L., AABBDD) is an allopolyploid staple crop that originated from two hybridization events involving three diploid species [16]. Approximately 800,000 years ago, *Triticum urartu* (AA) hybridized with *Aegilops speltoides* (BB), forming wild emmer wheat. Wild emmer (*Triticum dicoccoides*, AABB) subsequently underwent prolonged selection and diversification, giving rise to cultivated emmer (*Triticum dicoccum*, AABB). Cultivated emmer later hybridized with *Aegilops tauschii* (DD) to produce common wheat [17,18,19]. Although starch synthesis-related genes have been extensively studied in many crops, their roles in wheat and its progenitors remain unclear. This study aims to characterize the genomic organization, phylogenetic relationships, expression patterns and genetic variations of SSRGs in wheat and its progenitors, thereby identifying candidates for functional validation and providing insights into wheat evolution from the perspective of starch synthesis.

## 2. Results

### 2.1. Genome-Wide Identification and Phylogenetic and Collinearity Analyses of SSRGs in Wheat

**Figure 1:**
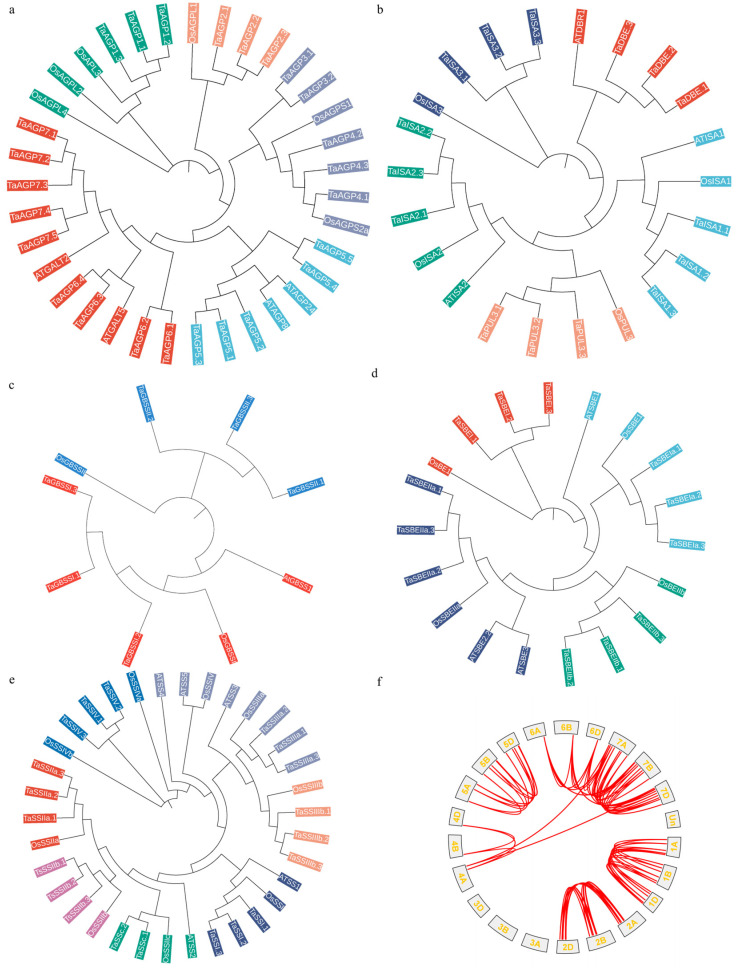
Phylogenetic tree of SSRG families and intraspecies collinearity. (**a**): The *AGP* gene family is divided into five branches, each represented by a different color. (**b**): The *DBE* gene family is divided into five branches, each represented by a different color. (**c**): The *GBSS* gene family is divided into two branches, each represented by a different color. (**d**): The *SBE* gene family is divided into four branches, each represented by a different color. (**e**): The *SSS* gene family is divided into seven branches, each represented by a different color. (**f**): Collinearity analysis of SSRGs across different wheat chromosomes. The A, B, and D sub-genomes that constitute the hexaploid genome of common wheat and jointly contribute to its agronomic traits and environmental adaptability [20].

Through a genome-wide search using homologs from *Arabidopsis thaliana* and rice (*Oryza sativa*), we identified 78 putative starch synthesis-related genes (SSRGs) in wheat. Analysis of the encoded protein sequences showed that their lengths range from approximately 315 to 1629 amino acids, with molecular weights spanning from 33,941 to 183,057 Da, indicating substantial variability among wheat SSRGs at the protein level. The predicted isoelectric points range from 4.87 to 9.3, suggesting that these proteins may function in different biochemical environments. Instability index analysis identified 43 proteins with values < 40, indicating that these proteins are likely to remain stable over extended periods and continuously perform their functions. Additionally, in the hydrophilicity analysis, 76 of the 78 proteins exhibited a Grand Average of Hydropathicity (GRAVY) score < 0, whereas only TaAGP5.4 and TaAGP5.5 had scores > 0. These results indicate that except for these two proteins, the proteins encoded by wheat SSRGs are predominantly hydrophilic. Chromosomal mapping showed that the 78 genes are distributed across 18 chromosomes. The distribution pattern further indicates that starch synthesis-related genes are mainly concentrated on chromosomes 1, 2, 5, and 7, which together account for 89.7% of the total genes (Figure 1f; Tables S2 and S14).

**Figure 2:**
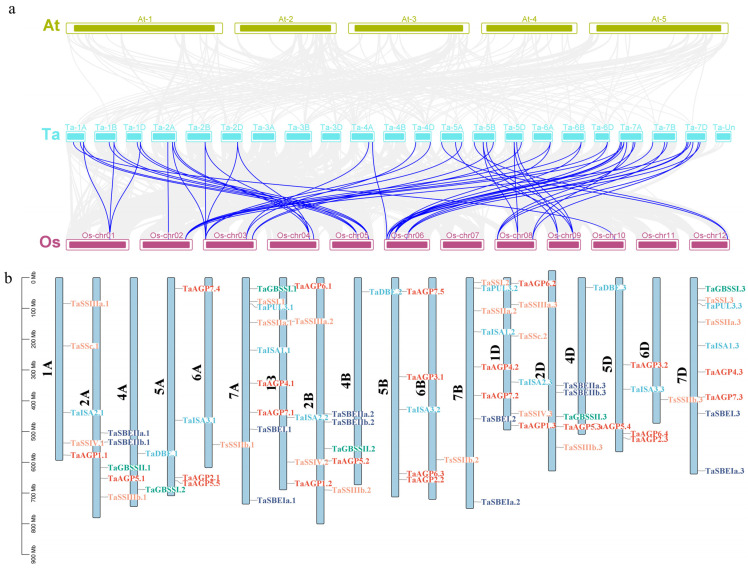
(**a**): Collinearity analysis among *Arabidopsis thaliana*, wheat, and rice. At: *Arabidopsis thaliana*; Ta: wheat; and Os: rice. (**b**): Chromosome distribution. Red represents the *AGP* family, blue represents the *DBE* family, green represents the *GBSS* family, orange represents the *SSS* family, and blue-violet represents the SBE family.

Phylogenetic analyses were conducted separately for genes encoding starch biosynthetic enzymes according to their functional classification. The results showed that genes belonging to the AGP and DBE families formed five clades in the phylogenetic tree; GBSS family genes clustered into two clades; SBE family genes were divided into four clades; and SSS family genes formed seven distinct clades (Figure 1a–e). The analysis further showed that 76 of the 78 genes have undergone sequence duplication events, with duplications observed between chromosomes 6 and 7 (Figure 2b). In addition, comparative collinearity analysis of SSRGs among wheat, rice, and *Arabidopsis thaliana* revealed no collinearity between wheat and *Arabidopsis thaliana*, whereas 56 collinear gene pairs were identified between wheat and rice (Figure 2a). These findings indicate that the starch synthesis genes in wheat and rice share a closer evolutionary relationship than those in *Arabidopsis thaliana*.

### 2.2. Collinearity, Conserved Motifs and Exon–Intron Structure Analysis

**Figure 3:**
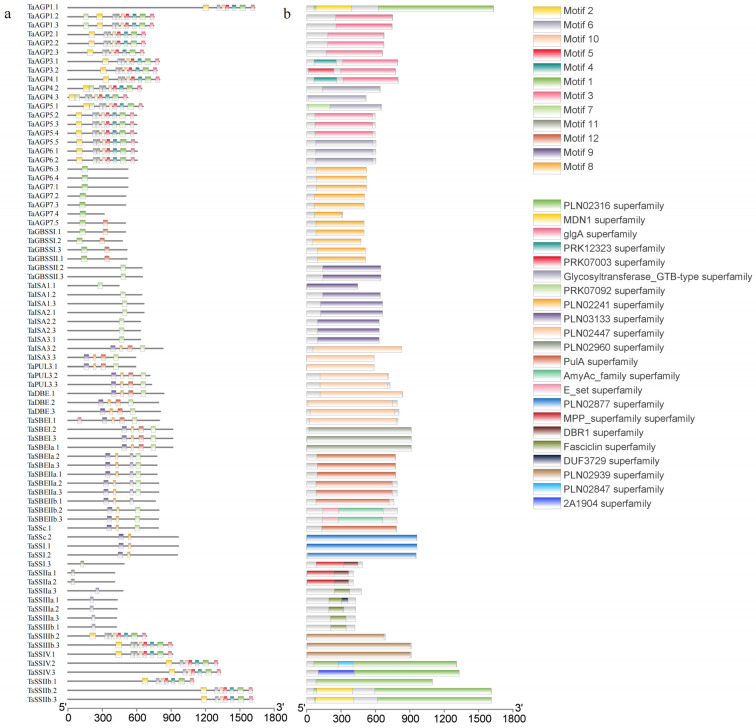
SSRG protein characteristics. (**a**): Conserved motifs; (**b**): conserved domains.

**Figure 4:**
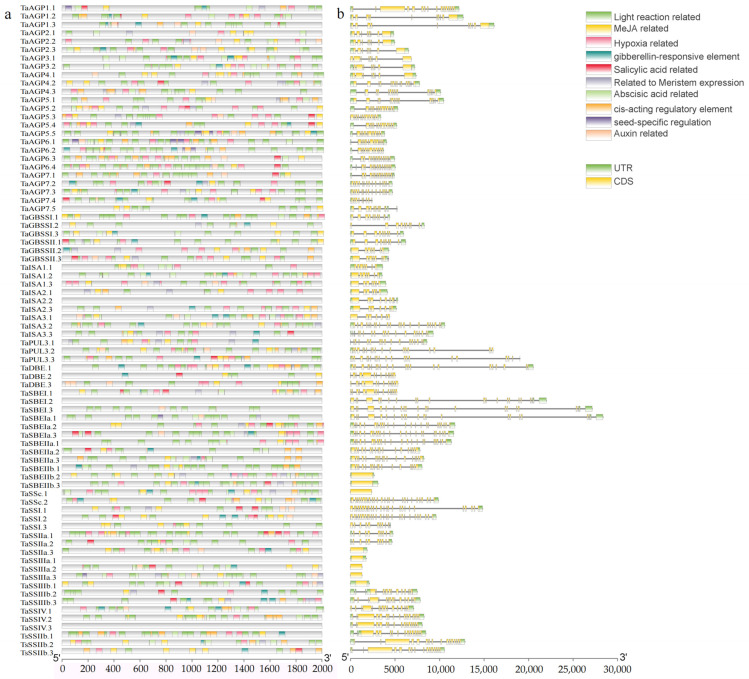
SSRG gene characteristics. (**a**): Cis-acting element; (**b**): gene structure.

Systematic analysis of 78 SSRGs identified 12 conserved motifs (Table S4). Among these, *TaSSI.1*, *TaSSI.2*, and *TaSSI.3* contained the highest number of motifs, whereas 23 genes possessed only one. Gene structure analysis showed that most SSRGs are split genes, with only eight lacking introns (Figure 3a). Conserved domains analysis (Figure 3b) identified 22 functional domains across the 78 sequences, classified into five enzyme types: AGPase (4 domains), SS (10), GBSS (2), SBE (2), and DBE (6). Notably, GBSS domains are identical to those of SS. Given the diverse roles of SS in starch synthesis, it was further subdivided into five functional classes. Analysis of cis-acting elements in the promoter regions of TaSSRGs revealed that methyl jasmonate, abscisic acid, light, and auxin-responsive elements are predominant (Figure 4a). These elements are associated with plant growth, development, stress responses, and energy regulation. Most starch synthesis genes also exhibited highly similar structural features, suggesting that gene structure may play a key role in regulating expression and influencing starch biosynthesis (Figure 4b).

### 2.3. Expression Profiles of SSRGs Based on RNA-Seq Data

**Figure 5:**
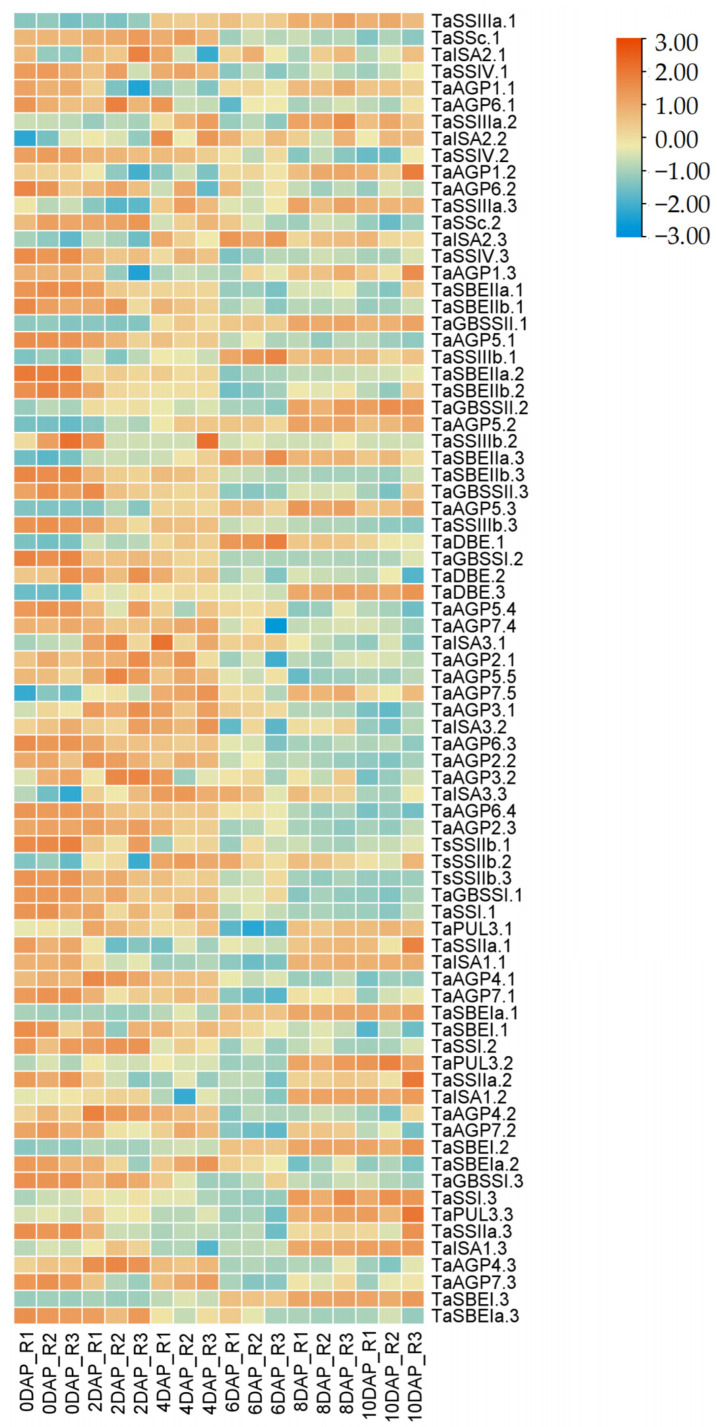
Transcript profiles of SSRGs. Expression profiles of SSRGs during early grain development; 0 DAP indicates 0 days after pollination, and subsequent time points follow accordingly.

To further infer SSRG function, spatiotemporal expression profiles were analyzed in wheat grains at 0, 2, 4, 6, 8, and 10 days post-flowering using public RNA-seq data (Figure 5). During the 0–4 days, SS, GBSS, and some AGPases showed relatively high expression. At 6 days, only *TaISA2s* and *TaAPG5s* remained highly expressed, while other SSRGs were downregulated. From 8 to 10 days, SBE and ISA exhibited predominant expression, with *TaSSIs*, *TaAGP4s*, and *TaSSIIIas* also showing high levels. These stage-specific expression patterns likely reflect functional specialization within the starch synthesis pathway, where different enzymes act at distinct developmental stages and locations.

### 2.4. Genetic Variation and Haplotype Analysis

**Figure 6:**
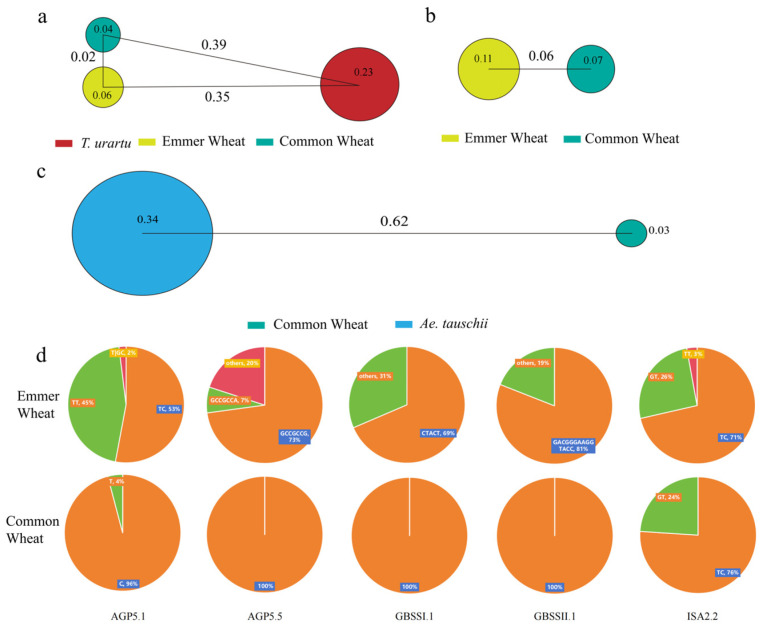
Genetic Characteristics of SSRGs. (**a**), (**b**), and (**c**) represent genetic differentiation and diversity among populations in the A, B, and D sub-genomes, respectively. Red represents *Triticum urartu*, yellow represents emmer wheat, dark blue represents common wheat, and light blue represents *Aegilops tauschii*. Nucleotide diversity (π) was used to assess intra-population genetic diversity, while the fixation index (Fst) was used to measure genetic differentiation among populations [21]. (**d**): Haplotype analysis of five genes (*AGP5.1*, *AGP5.5*, *GBSSI.1*, *GBSSII.1*, and *ISA2.2*) between emmer wheat and common wheat.

Based on publicly available re-sequencing data from wheats of different ploidy levels, genetic variations in the identified SSRGs were extracted, and the genetic differentiation index and diversity of each gene were calculated. As shown in Figure 6, overall genetic diversity decreased in the following order: *Aegilops tauschii*, *Triticum urartu*, emmer wheat, and common wheat. This trend is further supported by the distribution of the genetic differentiation index among populations (Figure 6a–c, Tables S6 and S7).

Considering both the genetic differentiation index and genetic diversity, the π values of the *Triticum urartu* population in the A sub-genome differed significantly from those of emmer wheat and common wheat, resulting in correspondingly high Fst values. In contrast, between emmer wheat and common wheat, only four genes showed significantly different π values, while the values of the remaining genes were relatively similar. Consequently, the genes with Fst values >0.05 were limited to *TaGBSSI.2*, *TaISA2.1*, and *TaSBEIIb.1*.

In the B sub-genome, the diversity of emmer wheat generally showed higher diversity than that of common wheat. Moreover, compared with the A sub-genome, the B sub-genome exhibited even higher overall diversity. Consequently, there were more genes in the B sub-genome with Fst > 0.05. In the D sub-genome, a significant difference in genetic diversity was observed between *Aegilops tauschii* and common wheat, with most starch-synthesis genes in common wheat showing relatively low diversity. Among the 27 genes in the D sub-genome, only *TaAGP5.3* had an Fst value of 0, whereas all other SSRGs had Fst > 0.05. Haplotype analysis of 78 SSRGs in durum wheat and common wheat showed that SSRG haplotypes in durum wheat were highly dispersed, with no major haplotypes. Only five genes (*AGP5.1*, *AGP5.5*, *GBSSI.1*, *GBSSII.1*, and *ISA2.2*) showed relatively conserved haplotypes. In contrast, most SSRG haplotypes in common wheat were mainly distributed into 2–3 types, typically with one major haplotype, and in a few cases, two.

### 2.5. Comparative Analysis of Amylose Between Emmer and Common Wheat

**Figure 7:**
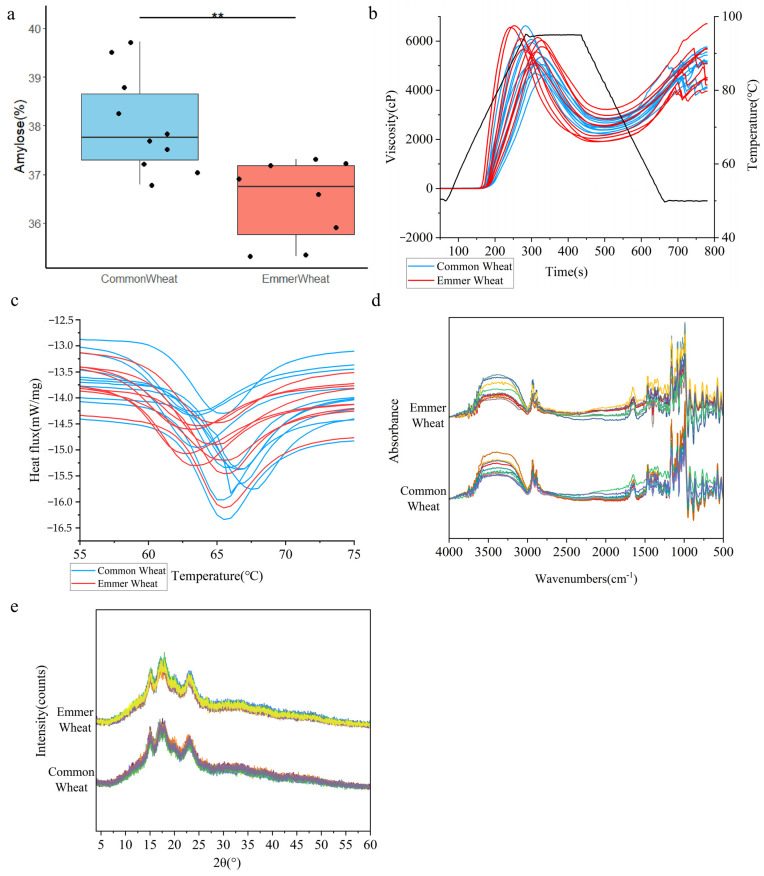
Analysis of physicochemical properties and structural characteristics of starch. (**a**): Comparative analysis of amylose content in wheat; (**b**): Starch viscosity; (**c**): Thermodynamic properties; (**d**): FTIR spectra; (**e**): X-ray diffraction. In panels a-c, blue represents common wheat and red represents emmer wheat. Panels d and e illustrate differences in starch crystalline structure between emmer wheat and common wheat at different wavebands and diffraction angles. To ensure the differentiation of differences between different samples, different colors were used for each sample in (**d**,**e**), but the comparison was made between the changing trends of emmer wheat and common wheat. Note: 0.01 (**).

Amylose plays a pivotal role in determining starch quality [22]. Representative varieties were selected to characterize the amylose content of common wheat and emmer wheat, and the results are shown in Figure 7a. A comprehensive analysis revealed a significant difference between the two species. The starch content of common wheat typically ranged from 18% to 29% [23]. In this study, amylose content was relatively high across all samples, likely because the materials analyzed were previously extracted starch. Within populations, amylose content was more variable in common wheat and more uniform in emmer wheat. Overall, common wheat (38.51 ± 1.80%) exhibited higher amylose content than emmer wheat (36.95 ± 1.59%).

### 2.6. Analysis of Viscosity, Thermodynamic and Crystal Properties of Wheat Starch

The viscosity properties of common and emmer wheat starches showed no significant overall differences. Key parameters were ranked as follows: peak viscosity (5588.86 ± 567.322 cP) > final viscosity (5068 ± 801.098 cP) > setback value (2539.45 ± 496.975 cP) > trough viscosity (2528.55 ± 453.476 cP) > breakdown value (3060.31 ± 836.121 cP). The average peak time was recorded at 5.09 ± 0.493 min. Common wheat starch showed a slightly delayed peak time but greater viscosity stability, whereas emmer wheat starch exhibited higher peak viscosity and breakdown values, indicating greater viscosity variability.

Common wheat starch exhibited higher initial (61.2173 ± 1.71071 °C), peak (65.2933 ± 1.49863 °C) and final (75.119 ± 1.71983 °C) gelatinization temperatures. Significant differences were observed in the initial and peak temperatures (*p* < 0.01), and also in the final temperature (*p* < 0.05). This greater thermodynamic stability may be associated with higher amylose content.

X-ray diffraction [24] (Table S10, Figure 7e) showed A-type crystal structures in both starches with similar profiles. Common wheat starch had higher diffraction intensities at 15°, 17°, 18° and 23.5°, but lower at 20° (2.382 ± 1.123 vs. 3.511 ± 0.889 a.u.), with a significant difference. Crystallinity did not differ significantly, though it was slightly higher in common wheat starch (22.489 ± 1.552% vs. 22.086 ± 1.119%). Fourier transform infrared spectroscopy [25] (Table S11, Figure 7d) indicated no significant differences in absorption peak intensities. However, common wheat starch exhibited a higher granule order index (1045/1022), whereas emmer wheat starch had a superior double-helix index (1022/995).

### 2.7. Apparent Characteristics of Starch Granules

**Figure 8:**
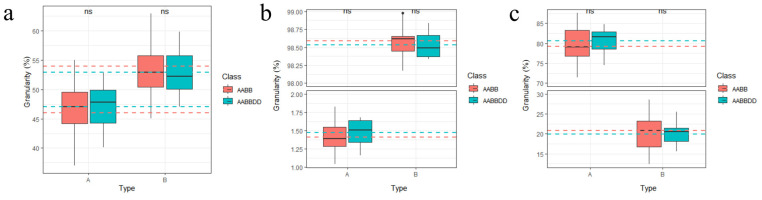
Starch granule characterization. (**a**): Surface area distribution; (**b**): Number distribution; (**c**): Volume distributio.; In panels (**a**–**c**), the red and blue dashed lines represent the average granule size of emmer wheat and common wheat, respectively. Type A and Type B denote A-type and B-type starch granules, respectively. The dashed lines in the figure represent the average values of their corresponding box plots.

A comprehensive particle-size analysis was performed on emmer and common wheat starch granules, classified as type A (>10 μm) and type B (<10 μm) [26]. Surface area, number, and volume distributions were systematically compared. As shown in Figure 8a–c and Table S12, no significant differences were observed in these distributions between the two wheat types. Emmer wheat exhibited a broader distribution range for surface area and volume, along with a more concentrated number distribution. Consistent with typical characteristics, type A granules were considerably fewer in number but larger in volume than type B granules, whereas type B granules contributed slightly more to surface area, reflecting the expected typical particle-size distribution pattern [27].

**Figure 9:**
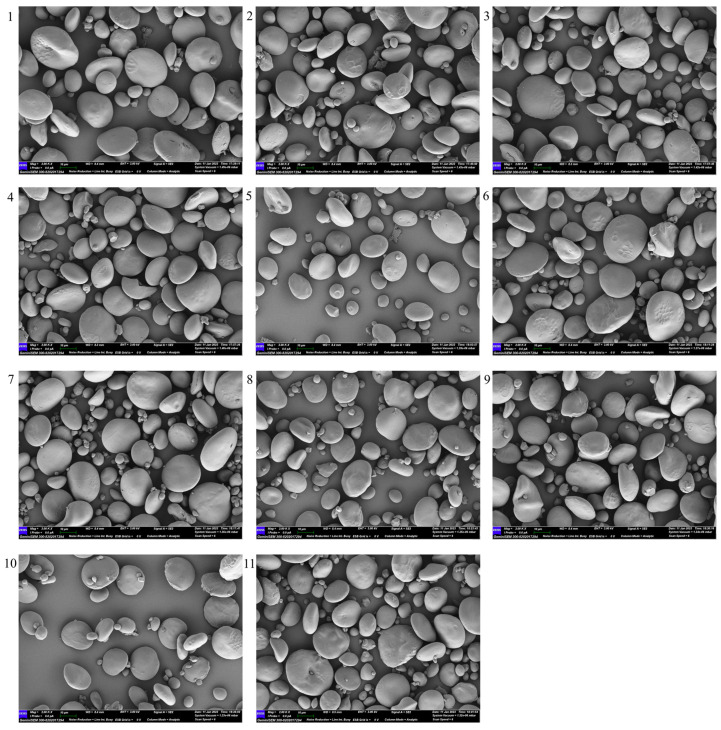
Common wheat electron microscope photos. 1–11 represent 11 different varieties of common wheat.

**Figure 10:**
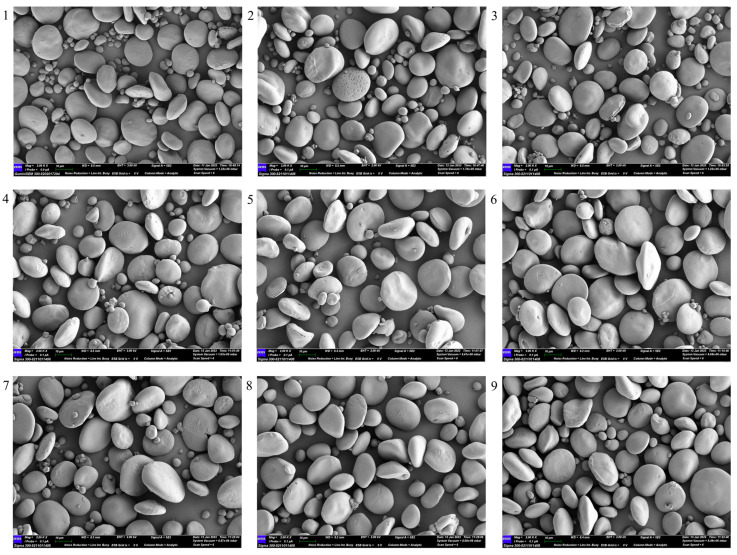
Emmer wheat electron microscope photos. 1–9 represent 9 different varieties of emmer wheat.

Scanning electron microscopy (SEM) was performed on 11 common wheat and 9 emmer wheat samples (Figure 9 and Figure 10) to characterize starch granule morphology. Granules from both wheat types were generally circular or elliptical, with a heterogeneous distribution of size and volume. Their surfaces were relatively smooth, with larger granules predominating and smaller ones interspersed. Fragmented and damaged granules were also observed. Overall, the particle-size characteristics and morphological features of starch granules were comparable between emmer and common wheat, with only minor differences in distribution range and concentration.

## 3. Discussion

The primary pathways of starch biosynthesis have been elucidated [28,29], and the genes encoding key starch synthases in model plants such as rice [30] and *Arabidopsis thaliana* [31,32] have been well documented. Although Gu [33] identified genes related to starch synthesis in wheat, their research primarily focuses on the overall starch synthesis pathway, lacking a concentrated investigation into the genes associated with starch synthesis in wheat protoplasts. Compared to Gu’s discovery, our study identified 46 genes related to starch synthesis that were identical to those previously reported, with consistent functional results. Furthermore, this study has newly discovered 32 additional genes associated with starch synthesis. Wheat starch synthesis is a complex, multi-stage process involving coordinated transcriptional regulation of enzyme genes, genetic variation, and environmental interactions [34]. Here, we performed the first genome-wide identification of wheat SSRGs and conducted a comprehensive analysis integrating their evolutionary history, genetic diversity, and phenotypic effects in tetraploid and hexaploid wheat. Our results reveal patterns of expansion, conservation, and divergence within this gene family and identify conserved haplotypes associated with key starch quality traits, providing new insights into the evolution and regulation of starch synthesis during polyploidization.

Phylogenetic and synteny analyses showed that most SSRGs are homoeologous duplications, consistent with the evolutionary history of hexaploid wheat [18,35]. Three Wx genes (*TaGBSSI.1* (*Wx-A1a*), *TaGBSSI.2* (*Wx-B1a*), and *TaGBSSI.3* (*Wx-D1a*)) were initially reported on chromosome 7B and later reassigned following segmental exchange with chromosome 4A [36,37,38]. Our synteny results further support polyploidization-driven duplication as the primary mechanism of gene family expansion. Additionally, *SSIIb* (chromosome 6) and *SSIIa* (chromosome 7) form a paralogous pair with clear sequence similarity, suggesting duplication prior to polyploidization. Protein structural analysis showed that conserved motifs and domain architectures across all genes align with established functional classifications.

Analysis of cis-acting elements in the promoter regions supports their regulatory roles in starch biosynthesis and validates the reliability of gene identification and classification. Gene expression profiling further clarifies functional timing. The heatmap shows that genes highly expressed during early grain development mainly belong to AGPase, SS, SBE, and GBSS, which drive starch initiation and elongation. In contrast, genes involved in branch trimming and structural modification (DBE, ISA, and PUL) peak during mid-to-late stages. These temporal expression patterns are consistent with their biological roles: early-stage enzymes mediate starch chain synthesis, whereas later-stage enzymes refine and remodel amylopectin structure [39,40,41].

Given the genomic complexity of wheat as an allohexaploid, we further analyzed the genetic diversity of SSRGs among diploid, tetraploid, and hexaploid ancestral species to characterize their features along wheat’s evolutionary trajectory. The results show that genetic diversity and population differentiation indices follow wheat’s ploidy evolution pattern: as chromosomal ploidy increases from diploid to hexaploid, genetic diversity declines. This trend is consistent with findings reported by Cheng et al. [42] for wheat populations with different ploidy levels. Haplotype analysis provides further evidence: starch synthesis-related genes in tetraploid emmer wheat show more dispersed haplotypes, whereas hexaploid common wheat contains a few highly conserved dominant haplotypes. This suggests that polyploidization, along with domestication and breeding, imposed strong selection for key starch quality traits, leading to genetic purification and fixation of superior alleles.

This comparative study demonstrates that the primary differences in starch composition between emmer and common wheat are characterized by variations in amylose content and gelatinization temperature, while the crystalline structure and granule morphology remain conserved.

The broader range and higher diversity of amylose content in common wheat can be attributed to its hexaploid genetic architecture. Common wheat possesses three Waxy (*Wx*) loci (*Wx-A1*, *Wx-B1*, *Wx-D1*), which encode GBSSI. In contrast, tetraploid emmer possesses only the *Wx-A1* and *Wx-B1* loci [43,44]. This triplicate set of loci provides a greater combinatorial potential for allelic variation, including null mutations that lead to partial or waxy phenotypes, thereby enhancing the diversity of amylose content [45,46]. In contrast, the more limited *Wx* complement in emmer restricts its amylose range [47].

The higher gelatinization temperature observed in common wheat starch is likely attributable to its elevated amylose content, which promotes the formation of more ordered crystalline regions that require greater thermal energy for disruption [48]. Furthermore, the hexaploid genome may influence the fine structure of amylopectin through the alleles of starch synthase, thereby further modulating thermal properties [49].

No significant differences were detected in the crystalline structure or granule morphology. Both species exhibited the typical A-type X-ray diffraction pattern and a bimodal granule size distribution characteristic of cereal starches [50]. These features appear to be highly conserved within the *Triticum* genus.

From an applied perspective, emmer starch with lower gelatinization temperature may be more suitable for moderate-temperature processing. In contrast, the broader amylose diversity found in common wheat provides greater flexibility for breeding programs aimed at specific starch functionalities, such as high resistant-starch genotypes for nutritional enhancement.

## 4. Materials and Methods

### 4.1. Identification of Starch Biosynthesis-Related Genes in Wheat

Genes associated with starch synthesis in *Arabidopsis thaliana* (https://www.arabidopsis.org/; 16 January 2025) and *Oryza sativa* (https://www.ricedata.cn/; 16 January 2025) were retrieved from their respective online databases. A BLAST (2.15.0+) [51] database was constructed using the proteome of the wheat cultivar Chinese Spring (https://ftp.ensemblgenomes.ebi.ac.uk/pub/plants/current/fasta/triticum_aestivum/pep/; 16 January 2025). Protein sequences of SSRGs from Arabidopsis and rice were aligned against this database, and hits with E-values < 1 × 10^−5^ were retained as candidate wheat SSRGs.

The chromosomal distribution of SSRGs was visualized using TBtools (TBtools-II) [52] based on the wheat annotation file. Physicochemical properties of the encoded proteins were analyzed via the online platform Expasy (https://web.expasy.org/protparam/; 16 January 2025).

### 4.2. Phylogenetic Relationship, Conserved Motifs, Prediction of Cis-Acting Components and Gene Structure Analysis

Protein sequences from *Arabidopsis thaliana*, *Oryza sativa*, and wheat were aligned using the MAFFT (v7.505) software [53] (Table S3). A phylogenetic tree was constructed with IQ-TREE (2.1.4-beta) using ModelFinder (MFP) to select the best-fit evolutionary model and 1000 bootstrap replicates [54].

Intraspecific BLAST analysis was first performed on the proteome of Chinese Spring wheat, followed by collinearity analysis using MCScanX (v1.0.0) [55] and visualization in TBtools. Pairwise comparisons among wheat, *Arabidopsis thaliana*, and rice proteomes were then analyzed with MCScanX, and the results were integrated and visualized using TBtools. The coding and non-coding regions of wheat starch synthesis-related genes were distinguished using the annotation file of Chinese Spring wheat from the Ensemble Plants database. The NCBI-CDD search module (https://www.ncbi.nlm.nih.gov/cdd; 16 January 2025) was used to analyze the conserved domains in these wheat SSRG protein sequences.

The sequence of 2000 bp upstream of the transcription start site was extracted as the promoter region of SSRGs. Potential cis-acting elements were predicted using PlantCARE (http://bioinformatics.psb.ugent.be/webtools/plantcare/html/; 11 March 2025), and the results were visualized, classified, and analyzed. Conserved protein motifs were identified using MEME Suite (https://meme-suite.org/meme/index.html; 11 March 2025), with parameters set to default except for a maximum of 12 motifs.

### 4.3. Expression Profile Analysis of Starch Biosynthesis-Related Genes Under Stresses and Grains

To analyze the expression patterns of wheat starch synthesis-related genes during early grain development and under stress conditions, the transcripts per million (TPM) values from project PRJNA780107 were obtained from the Triticeae Expression Database (TriticeaeExpDB; https://www.triticeaeexpdb.cn/ta_search.php; 11 March 2025). The acquired TPM values of SSRGs were normalized in TBtools and transformed using log2(x + 1) to ensure consistent data representation.

### 4.4. Genetic Variations Analysis of Starch Biosynthesis-Related Genes in Wheat

Publicly available resequencing data from different wheat ploidy groups were used to assess genetic variation. Variation map data (GVM000082) for AA, AABB, AABBDD, and DD populations were retrieved from the National Genomics Data Center (https://ngdc.cncb.ac.cn/gvm/; 11 March 2025). SSRG-related variants were extracted using VCFtools (Table S5). Genetic differentiation (Fst) and nucleotide diversity (π) were calculated using VCFtools (version 0.1.16) [56]. Evaluation across multiple window sizes (1 bp, 10 kb, 100 kb, and 1 Mb) [57] showed consistent differentiation patterns, with single-site analysis (1 bp) providing the highest resolution for detecting hotspots. Accordingly, both window and step sizes were set to 1 bp; Fst and π values represent averages across the A, B, and D sub-genomes. Genetic differentiation and diversity maps were generated using Adobe Illustrator (Adobe Illustrator CC 2018). Finally, haplotype analysis of the SSRGs was performed using the R package geneHapR (version 1.2.5) [58].

### 4.5. Comparison of the Starch Properties Between Emmer and Common Wheat

Eleven representative bread wheat varieties and nine emmer wheat varieties were selected (Table S13). All materials were grown at the Agricultural Station of Northwest A&F University (34°18′ N, 108°05′ E) under uniform field conditions. Starch was extracted using a modified wet method based on the Syahariza [59] protocol. The extracted starch was pretreated according to the specifications of a fully automatic amylose analyzer (AMS, Futura3; AMS Alliance, Frépillon, France) and analyzed in triplicate. Thermodynamic properties were measured using a differential scanning calorimeter (TA, Q2000; TA Instruments, New Castle, DE, USA). Nitrogen was used as a protective gas, and the temperature of the gelatinization furnace was increased from 30 °C to 105 °C at a rate of 10 °C·min^−1^ with three replicates. The viscosity attributes of starch were determined using a rapid visco-analyzer (Perten, RVA4500; Perten Instruments, Stockholm, Sweden). X-ray diffraction (XRD) and Fourier transform infrared (FTIR) analyses of wheat starch were performed following the methodology described by Wang et al. [25,60]. For XRD, samples were scanned using an X-ray diffractometer (PANalytical, PANalytical X’Pert Pro, Almelo, The Netherlands) over a 2θ range to determine relative crystallinity and crystal morphology. FTIR spectra were recorded using a Fourier transform infrared spectrometer (Thermo, Nicolet iZ-10; Thermo Fisher Scientific, Waltham, MA, USA) to assess structural features. Starch particle size was measured by wet analysis using a laser particle size analyzer (Matersizer 3000, Malvern Instruments Ltd., Malvern, UK), and granule morphology was observed by SEM. Imaging was performed according to Deng et al. [61]. Briefly, 100 mg of the sample was pretreated, sputter-coated with gold, and examined microscopically.

## 5. Conclusions

A total of 78 genes associated with wheat starch synthesis were successfully identified in this study, with some overlapping with previously reported genes. Phylogenetic analysis classified these SSRGs according to their distinct functional roles. However, their expression patterns during early grain development did not strictly align with these classifications. Notably, the SSRGs in common wheat are generally more conserved than those in emmer wheat. Comparative analysis showed that emmer and common wheat differed significantly in amylose content and thermodynamic properties, whereas no significant differences were detected in crystalline structure or granule morphology.

## Data Availability

The datasets supporting the conclusions of this article are included within the article and its additional files.

## Supplementary Materials

The following supporting information can be downloaded at: https://www.mdpi.com/article/10.3390/ijms27093876/s1.
